# Association of Eleven Common, Low-Penetrance Colorectal Cancer Susceptibility Genetic Variants at Six Risk Loci with Clinical Outcome

**DOI:** 10.1371/journal.pone.0041954

**Published:** 2012-07-27

**Authors:** Janelle M. Hoskins, Pei-Shi Ong, Temitope O. Keku, Joseph A. Galanko, Christopher F. Martin, Clint A. Coleman, Michelle Wolfe, Robert S. Sandler, Howard L. McLeod

**Affiliations:** 1 Division of Pharmacotherapy and Experimental Therapeutics, Institute for Pharmacogenomics and Individualized Therapy, Eshelman School of Pharmacy, University of North Carolina at Chapel Hill, Chapel Hill, North Carolina, United States of America; 2 Division of Gastroenterology and Hepatology, University of North Carolina at Chapel Hill, Chapel Hill, North Carolina, United States of America; 3 Center for Gastrointestinal Biology and Disease, Division of Gastroenterology and Hepatology, University of North Carolina at Chapel Hill, Chapel Hill, North Carolina, United States of America; 4 Division of Hematology and Oncology, University of North Carolina at Chapel Hill, Chapel Hill, North Carolina, United States of America; 5 The Lineberger Comprehensive Cancer Center, University of North Carolina at Chapel Hill, Chapel Hill, North Carolina, United States of America; Ohio State University Medical Center, United States of America

## Abstract

**Background:**

Low-penetrance genetic variants have been increasingly recognized to influence the risk of tumor development. Risk variants for colorectal cancer (CRC) have been mapped to chromosome positions 8q23.3, 8q24, 9p24.1, 10p14, 11q23, 14q22.2, 15q13, 16q22.1, 18q21, 19q13.1 and 20p12.3. In particular, the 8q24 single nucleotide polymorphism (SNP), rs6983267, has reproducibly been associated with the risk of developing CRC. As the CRC risk SNPs may also influence disease outcome, thus in this study, we evaluated whether they influence patient survival.

**Methodology/Principal Findings:**

DNA samples from 583 CRC patients enrolled in the prospective, North Carolina Cancer Care Outcomes Research and Surveillance Consortium Study (NC CanCORS) were genotyped for 11 CRC susceptibility SNPs at 6 CRC risk loci. Relationships between genotypes and patient survival were examined using Cox regression analysis. In multivariate analysis, patients homozygous for the CRC risk allele of rs7013278 or rs7014346 (both at 8 q24) were only nominally significant for poorer overall survival compared to patients homozygous for the protective allele (hazard ratio = 2.20 and 1.96, respectively; *P*<0.05). None of these associations, however, remained statistically significant after correction for multiple testing. The other nine susceptibility SNPs tested were not significantly associated with survival.

**Conclusions/Significance:**

We did not find evidence of association of CRC risk variants with patient survival.

## Introduction

Colorectal cancer (CRC) is the second most common cause of cancer-related death in the United States. Despite improvements in treatment modalities, the 5-year survival rate for CRC patients ranges from 10–90% [1]. This huge variation in clinical outcome is due, in part, to the fact that CRC is a heterogeneous disease comprising discrete subsets that evolve through multiple different etiologies. Both germline and somatic genetic alterations can be involved in the malignant transformation of normal colon cells. Extensive investigations have identified somatic mutations in *TP53* or *KRAS* that are involved in the progression of adenoma to CRC [2]–[5]. For germline mutations, high-penetrance changes in adenomatosis polyposis coli, mismatch repair, mothers against decapentaplegic homolog 4, bone morphogenetic protein receptor type IA and serine threonine kinase 11 genes have been reported to be associated with increased CRC susceptibility in 5% of the population [6] while the effects of combinations of low-penetrance variants remains largely elusive.

The completion of the Human Genome Project and the development of improved high-throughput genotyping techniques permit large scale interrogation of the genome, resulting in a better understanding of common polygenic disease. This progress has led to the common disease, common variant hypothesis, which suggests that a number of allelic variants found in more than 1–5% of the population genetically influences the susceptibility to common heritable diseases [7]. In line with this model, candidate gene analysis and multi-stage genome wide association studies (GWAS) have identified numerous single nucleotide polymorphisms (SNPs) across several chromosomes that are associated with an increased risk of CRC development [8]–[18]. In a case-control study involving 1807 patients and 5511 controls, Haiman and colleagues noted that rs6983267 on chromosome 8q24, a genomic region that contains few genes, was significantly linked to a higher predisposition of CRC in individuals of different ethnicities [10]. Several later reports confirmed rs6983267 as a low-penetrance risk marker for CRC [9], [12], [13], [16], [17]. Given this consistent observation and the frequent amplification of this region in CRC [10], further analysis of SNPs within this gene-poor area revealed a second tightly linked variant, rs10505477, as well as three other SNPs on 8q24, rs10808556, rs7014346 and rs7013278, as low-penetrance variants that also influence carcinoma formation [10], [13], [14], [15], [17].

Besides the SNPs on 8q24, Tomlinson et al identified rs16892766 on 8q23.3 as an additional risk variant [15]. Likewise, Zanke and colleagues found an association between rs719725 on 9p24 and CRC [17]. Subsequent investigation of this site by Poynter et al showed that individuals with the A/A genotype had a moderate increased risk of CRC compared to C/C individuals in population-based but not clinic-based families [13]. On the 10p14 and 11q23 loci, rs10795668 and rs3802842 were respectively shown to be related to disease occurrence [14], [15]. In addition, numerous other risk loci have been subsequently identified and mapped to 14q22.2 (rs4444235, BMP4) [18], 15q13 (rs4779584, rs10318, CRAC1) [11], 16q22.1 (rs9929218, CDH1) [18], 18q21 (rs4939827, rs12953717, rs4464148, SMAD7) [8], 19q13.1 (rs10411210, RPHN2) [18] and 20p112.3 (rs961253) [18].

Despite the increasing number of loci being identified to influence the risk of CRC development, to date, only a few studies have investigated the effects of these variants on disease outcome [19]–[22]. The findings of these studies however remained inconsistent on the relationship between these risk variants and CRC survival. Therefore, in the present study, we examined the prognostic significance of 11 CRC susceptibility SNPs at 6 CRC risk loci (rs6983267, rs10505477, rs7013278, rs7014346, rs719725, rs10795668, rs3802842, rs4779584, rs10318, rs4464148 and rs4939827, Table S1), using 583 patients with CRC from the prospective North Carolina (NC) Cancer Care Outcomes Research and Surveillance Consortium Study (CanCORS).

## Methods

### Study Population and Follow-Up Information

The study design of CanCORS has been described previously [23]. NC CanCORS assembled a prospective population-based cohort in 33 county areas of NC, USA. In this study, DNA samples from 583 eligible patients diagnosed with CRC between April 2003 and January 2005 were retrospectively genotyped. Patient demographics were obtained from a baseline patient survey. Detailed clinical information on primary site of tumor, American Joint Committee on Cancer (AJCC) stage and type of treatment provided were obtained from review of medical records and pathological reports of CRC diagnosis from the North Carolina Central Cancer Registry by trained abstractors within 6 months following diagnosis. Patients were followed up for a median of 3.5 years. Survival and mortality data was determined from a three-year phone survey of participants or next-of kin and further confirmed through ascertainment of death records through the social security death index (SSDI) using patients’ social security numbers. Mortality information or survival status was available for all patients genotyped in this study through the SSDI. Disease-free survival information was not available. This study was approved by the Institutional Review Board of the University of NC (IRB number: 04–08–60). Written informed consent was obtained from each participant.

### DNA Extraction and SNP Genotyping

Buffy coat was prepared from a blood sample collected from each study participant. DNA was extracted from buffy coat using Puregene kit (Qiagen, Valencia, CA, USA). In addition, DNA samples comprising of 94 European-Americans, 93 Han Chinese (Han people of Los Angeles) and 94 Mexican-Americans (Mexican-American community of Los Angeles) from Coriell (Coriell Institute for Medical Research, Camden, NJ, USA) as well as 86 African-Americans as previously described [24], were also used to confirm the reliability and reproducibility of the genotyping performed. All DNA samples were genotyped for 11 CRC susceptibility SNPs at 6 CRC risk loci using TaqMan allelic discrimination assays (Applied Biosystems, Foster City, CA, USA) (Table S1). In a total volume of 20 µL, reactions consisted of 2 µL of 10 ng/µL DNA, 0.5 µL of 20X TaqMan® SNP genotyping assays (Applied Biosystems), 10 µL of 2X TaqMan® Universal PCR Master Mix, No AmpErase UNG (Applied Biosystems) and 7.5 µL of water. PCR was performed with an initial denaturation step at 95°C for 10 min followed by 50 cycles of denaturation at 92°C for 15 s and annealing with extension at 60°C for 1 min. All PCR reactions were performed on a Bio-Rad Tetrad 2 Thermal Cycler (Bio-Rad, Hercules, CA, USA). The fluorescence intensities of the samples were measured before and after PCR using an Applied Biosystems 7500 Real-Time PCR System (Applied Biosystems). Data obtained was analyzed and genotypes assigned using 7300 System SDS Software, version 1.4 (Applied Biosystems). Genotype allocation was performed blinded to patients’ clinical data. Genotyping was successful in >96% of samples. For the samples from the four additional populations, all genotypes were in agreement with that reported in the Hapmap (www.hapmap.org). No discordance in genotype or allele frequencies was observed between the European-American or African-American patient samples with the respective European-Americans or African-Americans in the additional populations genotyped.

### Statistical Analysis

All polymorphisms were examined for deviation from Hardy-Weinberg equilibrium (HWE) using χ^2^ test. Clinical and biological variables were examined for associations with individual SNPs using Fisher’s Exact Test. Univariate and multivariate survival analyses were carried out using Cox regression analysis to evaluate associations between genetic variants and overall survival. Multivariate models were adjusted for age, ethnicity, gender, disease stage, site of tumor and treatment administered. For each SNP, the risk allele was defined as the allele previously established in the literature to confer a risk of CRC while the other allele was considered the protective allele. Homozygosity for the protective allele served as a reference genotype for regression analysis and was assigned a hazard ratio (HR) of 1.0. A *P*<0.05 was considered significant. Multiple correction was performed using the conservative Bonferroni method. All data was analyzed using SAS statistical analysis software version 9 (SAS Institute Inc, Cary, NC, USA).

**Table 1:** Demographic, Histopathological and Clinical Characteristics of Study Patients.

		Patients
Characteristics		N (%)
**Patients**		583
**Age, years**	Median (range)	65.0 (26.0–93.0)
**Gender**	Male	304 (52.1)
	Female	279 (47.9)
**Ethnicity**	European-American	472 (81.0)
	African-American	111 (19.0)
**Stage**	1	161 (27.6)
	2	160 (27.4)
	3	165 (28.3)
	4	68 (11.7)
	Missing	29 (5.0)
**Tumor Site**	Colon	261 (44.8)
	Rectum	241 (41.3)
	Missing	81 (13.9)
**Treatment - Surgery**	Yes	573 (98.3)
	No	8 (1.4)
	Missing	2 (0.3)
**Treatment – Chemotherapy**	Yes	297 (50.9)
	No	279 (47.9)
	Missing	7 (1.2)

## Results

### Patient Characteristics

Table 1 summarizes the baseline characteristics of patients in this study. The median age of patients was 65.0 years old and 47.9% were female. Among the 583 patients, 81.0% were European-Americans and 19.0% were African-Americans. At the time of diagnosis, the proportions of study participants classified to AJCC stages 1, 2, 3 and 4 were 27.6%, 27.4%, 28.3% and 11.7% respectively. In this cohort, the proportion of patients with colon cancer or rectal cancer was comparable. The majority of patients (98.3%) received surgical treatment while chemotherapy was administered to 50.9% of the study population.

### Low-Penetrance CRC Susceptibility Genotypes

The distribution of genotypes in this patient cohort is presented in Table S2. For this group of patients, no deviation from HWE was detected among European- or African-American patients for any of the SNPs. The allele frequencies for all SNPs were similar to frequencies of European- (CEU) and African-American populations (ASW) reported in HapMap (www.hapmap.org). Genotype distributions varied between European- and African-American patients for all SNPs (*P<*0.05) except rs7014346 and rs3802842 (Table S2).

### Relationships Between Low-Penetrance CRC Susceptibility SNPs and Clinicopathological Features of CRC Patients

No significant differences were observed between the SNPs with gender, tumor site, surgical intervention or chemotherapy administration (Table S2). For age of CRC diagnosis, apparent differences in genotype distribution were only found for rs10795668 (*P = *0.005; Table S2). It was observed that the prevalence of the A/A genotype for this SNP decreases in patients older than 50 years. In addition, stage of disease was significantly related to rs4464148 and rs4939827 genotypes (*P<*0.05; Table S2). C and T alleles, the risk allele for rs4464148 and rs4939827 respectively, were shown previously to be associated with an increased risk for CRC [8], [21]. Surprisingly in the present study, more patients homozygous for the risk allele for both SNPs presented with earlier stage cancer (stages 1–2) at the time of diagnosis, suggesting that these alleles are protective (Table S2).

### Relationships Between Low-Penetrance CRC Susceptibility SNPs and Overall Survival

To test whether the germline variants underlie differences in overall survival in CRC patients, we performed both univariate and multivariate survival analyses. In univariate analysis, a significant difference in survival was only observed in patients with the A/C genotype compared to the A/A genotype for rs3802842 (*P*<0.05; Table 2). This difference was not significant in a multivariate model (*P*>0.05; Table 2). In multivariate analysis, patients carrying two risk alleles (T/T) for rs7013278 had reduced survival compared with patients homozygous for protective allele (C/C) (HR = 2.20; 95% CI = 1.24–3.91; *P* = 0.01; Table 2). Likewise, patients homozygous for the CRC risk allele of rs7014346, A/A, showed inferior overall survival to G/G patients (HR = 1.96; 95% CI = 1.08–3.52; *P* = 0.03; Table 2). The moderate level of significance for these two SNPs was not maintained upon correction for multiple testing. No additional significant association was observed between the rest of the SNPs studied and survival (Table 2).

**Table 2:** Associations between genotypes and overall survival of study patients.

		Univariate			Multivariate#		
SNP and Genotype	Risk Allele	Hazard Ratio	95% CI	*P*	Hazard Ratio	95% CI	*P*
**rs6983267**							
T/T	G	1.00	–	–	1.00	–	–
G/T		0.85	0.51–1.41	*0.53*	1.07	0.59–1.93	*0.83*
G/G		1.17	0.71–1.92	*0.54*	1.35	0.73–2.51	*0.35*
**rs10505477**							
G/G	A	1.00	–	–	1.00	–	–
A/G		0.90	0.55–1.48	*0.68*	1.14	0.64–2.06	*0.65*
A/A		1.21	0.74–1.98	*0.46*	1.38	0.74–2.55	*0.31*
**rs7013278**							
C/C	T	1.00	–	–	1.00	–	–
C/T		0.94	0.63–1.40	*0.76*	1.06	0.66–1.71	*0.81*
T/T		1.55	0.94–2.56	*0.08*	2.20	1.24–3.91	*0.01* *
**rs7014346**							
G/G	A	1.00	–	–	1.00	–	–
A/G		0.89	0.60–1.31	*0.56*	1.04	0.67–1.64	*0.84*
A/A		1.33	0.78–2.27	*0.29*	1.96	1.08–3.52	*0.03* *
**rs719725**							
C/C	A	1.00	–	–	1.00	–	–
A/C		1.29	0.69–2.39	*0.43*	0.93	0.47–1.86	*0.84*
A/A		1.07	0.57–2.01	*0.84*	0.80	0.39–1.62	*0.53*
**rs10795668**							
G/G	A	1.00	–	–	1.00	–	–
A/G		0.79	0.54–1.16	*0.23*	0.82	0.51–1.31	*0.40*
A/A		0.54	0.25–1.17	*0.11*	0.82	0.37–1.83	*0.62*
**rs3802842**							
A/A	C	1.00	–	–	1.00	–	–
A/C		1.53	1.05–2.23	*0.03* *	1.25	0.80–1.93	*0.33*
C/C		0.95	0.50–1.79	*0.88*	1.03	0.52–2.03	*0.94*
**rs10318**							
C/C	T	1.00	–	–	1.00	–	–
C/T		1.10	0.74–1.64	*0.63*	1.20	0.73–1.90	*0.50*
T/T		0.50	0.12–2.03	*0.33*	0.62	0.15–2.55	*0.50*
**rs4779584**							
C/C	T	1.00	–	–	1.00	–	–
C/T		0.92	0.63–1.34	*0.65*	0.72	0.45–1.14	*0.16*
T/T		1.02	0.52–1.98	*0.96*	1.01	0.46–2.22	*0.98*
**rs4464148**							
T/T	C	1.00	–	–	1.00	–	–
C/T		0.78	0.54–1.15	*0.21*	0.74	0.48–1.16	*0.19*
C/C		0.95	0.50–1.80	*0.87*	1.39	0.67–2.90	*0.38*
**rs4939827**							
C/C	T	1.00	–	–	1.00	–	–
C/T		0.90	0.60–1.34	*0.59*	0.82	0.52–1.30	*0.40*
T/T		0.82	0.50–1.36	*0.44*	0.81	0.45–1.48	*0.50*

# Potential confounding variables such as age, gender, ethnicity, stage, and treatment received were included in multivariate modeling

* Statistically significant at *P*<0.05.

## Discussion

The present study was performed to examine the prognostic significance of 11 common, low-penetrance genetic variants at 6 CRC loci that have been previously reported to predispose individuals to CRC [8]–[17]. Although we found marginal significance between two SNPs, rs7013278 and rs7014346 (HR = 2.20, *P* = 0.01 and HR = 1.96, *P* = 0.03 respectively), with inferior CRC survival by multivariate regression analysis, none of these variants showed study-wide association with survival after correction for multiple testing. It therefore highlighted that these known CRC risk variants, do not play a role in influencing CRC mortality, which is in agreement with the findings from three earlier studies [19]–[21].

So far, several prior studies have investigated the association between CRC susceptibility variants with disease progression and survival. Gruber and colleagues were the first to report no correlation between rs10505477 with CRC survival in a predominantly Jewish population [19]. In this study, a HR of 1.09 (CI = 0.89–1.32) was found between carriers of risk allele versus non carriers [19] Subsequently, Cicek and colleagues evaluated the influence of 5 other low- penetrance CRC risk markers (rs6983267, rs13254738, rs16901979, rs1447295, DG8S737) on the survival of 460 cases of stages II and III, Caucasian, CRC patients who were the participants of a phase III adjuvant therapy study [20]. While they observed a trend of decreased survival rate with the presence of the rare risk variants (HR for rs6983267 = 1.00, CI = 0.79–1.27; HR for rs13254738 = 1.14, CI = 0.91–1.43; HR for rs16901979 = 1.34, CI = 0.86–2.09; HR for rs1447295 = 1.11, CI = 0.79–1.55; HR for DG8S737 = 1.57, CI = 0.85–2.90) using a log additive model, none of the associations were statistically significant [20]. In a later study by Tenesa et al, no association was found between 10 risk variants and all-cause or CRC-specific mortality after adjustment for AJCC stage, age and sex in 2838 AJCC stages I-IV CRC patients of Scottish Ancestry [21]. Likewise, we observed a similar lacked of correlation between rs10505477 (HR using multivariate analysis = 1.38, CI = 0.74–2.55) with survival which is in agreement with the finding by Gruber et al (HR = 1.09, CI = 0.89–1.32) [19]. For rs6983267, there was again no association with CRC survival in our study (HR using multivariate analysis = 1.35, CI = 0.73–2.51). The HR for this SNP is in similar direction and again in agreement with previous reports by Cicek et al (HR = 1.00, CI = 0.79–1.27) and Tenesa et al (HR = 1.03, CI = 0.94–1.13) [20], [21]. Furthermore, for 4 other SNPs (rs10795668, rs3802842, rs4779584 and rs4939827), the HRs obtained by Tenesa and colleagues were 0.98 (CI = 0.90–1.08), 0.93 (CI = 0.85–1.02), 0.95 (CI = 0.85–1.06) and 1.05 (CI = 0.96–1.15) respectively [21]. This result is in congruent with our study with HRs for rs10795668, rs3802842, rs4779584 and rs4939827 to be 0.82 (CI = 0.37–1.83), 1.03 (CI = 0.52–2.03), 1.01 (CI = 0.46–2.22) and 0.81 (CI = 0.45–1.48) respectively, with the range of CI values for these SNPs overlapping between the two studies. This data thus reinforce the lack of evidence for the involvement of these variants with CRC outcome. In a recent study by Xing et al, the influence of 8 GWAS associated SNPs with CRC recurrence and survival was investigated in 465 Han Chinese CRC patients [22]. Two SNPs, rs4779584 (HR = 0.33, CI = 0.15–0.72 for homozygous carriers of the wild type allele (T), which is also the risk allele in GWAS, versus those who are homozygous or heterozygous carriers of the variant allele (C), the protective allele in previous GWAS) and rs10795668 (HR = 0.55, CI = 0.30–1.00 for homozygous carriers of the wild type allele (G), which is the protective allele in previous GWAS, versus those who are homozygous or heterozygous carriers of the variant allele (A), the risk allele in previous GWAS), were significantly associated with reduced risk of death and tumor recurrence respectively using a dominant genetic model [22]. For rs4779584, the result of this study (HR = 1.01, CI = 0.46–2.22) and that of Tenesa et al (HR = 0.95, CI = 0.85–1.06) [21] showed no significant influence of the risk allele of this SNP with CRC survival. This deviates from the findings of Xing et al [22]. A plausible explanation for this deviation is a much higher frequency of the risk allele among the Han Chinese compared to the population in this study and that of Tenesa and colleagues [21], which were predominately of European ancestry. While the allele frequencies of rs4779584 in the Han Chinese CRC patients were not directly reported in the paper by Xing et al [22], a calculation of the allele frequencies of this population based on the genotype of patients presented showed that the calculated allele frequencies (C = 0.19 and T = 0.81) were congruent with that from the Chinese populations in the Hapmap (CHB: C = 0.18 and T = 0.82; CHD: C = 0.16 and T = 0.84) and the Han Chinese population that we have previously genotyped (C = 0.17 and T = 0.83) as part of the four additional populations used for genotyping control. Based on the data from Hapmap and our data of the four additional populations genotyped (data not shown), the allele frequency for the risk allele (T) of rs4779584 was highest in the Han Chinese, followed by African-American and lastly European-American. For rs10795668, HR for overall survival in our study and that by Tenesa et al [21] were 0.82 (CI = 0.37–1.83) and 0.98 (CI = 0.90–1.08) respectively. This divergence in study results to that of Xing et al is likely attributed to methodological differences between studies rather than due to differences in allele frequencies as the study by Xing et al [22] evaluated CRC recurrence while our study and that by Tenesa et al [21] evaluated overall survival and overall mortality respectively.

This inconsistency in findings between reported studies is not surprising as the mechanisms by which these variants alter the risk of CRC is not fully understood and it is still unclear how they might influence tumor progression and patient survival. Currently, the potential functional effects of these SNPs have been most widely investigated for the variants at the 8 q24 gene locus. Since this locus is not known to encode for any gene, it was thus conceived that the variants found here may either lie within promoters or enhancer elements that can affect the transcription of genes outside this locus [10]. However, in a study using the UCSC Browser and VISTA Enhancer database, rs7013278 and rs7014346, which showed marginal significance in our study prior to Bonferroni correction, were not found in segments containing putative enhancers or in predicted regions of regulatory potential [25]. On the other hand, rs6983267 was found to lie within a putative enhancer element that binds TCF4, a transcription factor that interacts with ß-catenin to activate the transcription of Wnt target genes, which are activated in most CRCs [26], [27]. Additionally, some reports have shown that rs6983267 has a long range physical interaction with a promoter region of MYC in colorectal cancer cell lines [26], [28]. Despite this, no association between the risk allele of rs6983267 and MYC expression levels has been found in normal and cancerous colon tissues [17], [26], [27]. Therefore, the functional consequence of rs6983267 remains uncertain.

For rs10795668 at 10 p14, rs3802842 at 11 q23 and rs 4779584 at 15 q13, a systematic search by Niittymaki and colleagues failed to show any association of these SNPs with predicted enhancer or regulatory elements [29]. Further investigation using tumor samples again showed a lack of allelic imbalance between the risk allele of these SNPs with CRC, prompting the authors to conclude that these risk variants were unlikely somatically selected for neoplastic progression [29]. While the functional effects of these susceptibility SNPs remain to be further validated, the results of these functional studies to date support our finding that the majority of low-penetrance CRC variants are involved in initiation rather than progression of CRC.

The strength of this study is that the patients were drawn at random from 33 county areas in central and eastern NC. These regions include urban and rural areas and as such the subjects are diverse with respect to race and socioeconomic status. They are therefore more representative of a true CRC population sample compared with other studies that only include patients from a few institutions and thus have highly selected populations.

There are however some limitations in our study. Firstly, the limited sample sizes of certain stages prevented a more detailed subgroup analysis. At such, it is possible that associations restricted to patients from certain stages may have been missed. Secondly, the median follow-up period of 3.5 years may be too short especially for patients with stages I and II disease. This could have in turn resulted in a lower event rate when data from all four stages of patients were analysed together and hence led to the marginal association observed. Thirdly, information on disease free survival data which may be a better prognostic measure compared to overall survival is not available in our study. Fourthly, while we made a rigorous effort to take into consideration important clinical variables such as age, sex, ethnicity, stage of disease, site of tumor and type of treatment that may influence CRC survival in our data analysis, we however did not have information on patients’ mismatch repair status. This may have led to the combination of different types of CRC in the same group for analysis, thereby biasing the hazard estimates obtained. Lastly, the sample size of this study is moderate. This may make it underpowered to detect the association of the genetic variants with survival outcomes for two SNPs (rs719725 and rs10318) due to low allele frequencies in our population or for SNPs with small effects on survival. Thus, for rs719725 and rs10318, the results for these SNPs should be interpreted cautiously. Nonetheless, the results of this study will augment the findings of earlier studies, allowing for future meta-analysis that can further improve our understanding of the effects of these rare variants on CRC progression.

In conclusion, we observed no association between 11 CRC susceptibility variants at 6 CRC risk loci with disease outcome in our study population, suggesting little influence of these SNPs on CRC progression.

## Supporting Information

Table S1 **Chromosomal loci of low-penetrance CRC susceptibility SNPs tested, their susceptibility risk allele and TaqMan® SNP genotyping assay identifiers.**
(DOCX)

Table S2 **Distribution of patients’ clinicopathological characteristics according to genotypes for individual SNP.**
(DOCX)
