# Supplementary material for: Association of Eleven Common, Low-Penetrance Colorectal Cancer Susceptibility Genetic Variants at Six Risk Loci with Clinical Outcome
Source: PLoS One. 2012 Jul 27;7(7):e41954. doi: 10.1371/journal.pone.0041954 (PMC3407042; doi:10.1371/journal.pone.0041954)
Supplement: Table S1 — Chromosomal loci of low-penetrance CRC susceptibility SNPs tested, their susceptibility risk allele and TaqMan® SNP genotyping assay identifiers. (DOCX) [file pone.0041954.s001.docx]

**Table S1.** Chromosomal loci of low-penetrance CRC susceptibility SNPs tested, their susceptibility risk allele and TaqMan® SNP genotyping assay identifiers.

| **dbSNP reference SNP identifier** | **SNP** | **Chromosomal Location** | **Chromosomal Position**# | **Taqman^®^ SNP genotyping Assay I.D.** |
| --- | --- | --- | --- | --- |
| rs6983267 | G>T | 8q24 | 128482487 | [C_29086771_20](javascript:showAssayDetails('https://products.appliedbiosystems.com:443/ab/en/US/adirect/ab?cmd=ABAssayDetailDisplay&assayID=C__29086771_20&Fs=y');) |
| rs10505477 | A>G | 8q24 | 128476625 | C_29809139_20 |
| rs7013278 | C>T | 8q24 | 128484074 | [C_29086773_10](javascript:showAssayDetails('https://products.appliedbiosystems.com:443/ab/en/US/adirect/ab?cmd=ABAssayDetailDisplay&assayID=C__29086773_10&Fs=y');) |
| rs7014346 | A>G | 8q24 | 128493974 | C_29086780_10 |
| rs719725 | A>C | 9q24 | 6355683 | [C_616037_10](javascript:showAssayDetails('https://products.appliedbiosystems.com:443/ab/en/US/adirect/ab?cmd=ABAssayDetailDisplay&assayID=C____616037_10&Fs=y');) |
| rs10795668 | A>G | 10p14 | 8741225 | C_1779559_10 |
| rs3802842 | A>C | 11q23 | 110676919 | C_2750382_10 |
| rs4779584 | C>T | 15q13 | 30782048 | C_28019826_10 |
| rs10318 | C>T | 15q13 | 30813271 | C_12070332_20 |
| rs4464148 | C>T | 18q21 | 44713030 | C_27989234_10 |
| rs4939827 | C>T | 18q21 | 44707461 | [C_27913406_10](javascript:showAssayDetails('https://products.appliedbiosystems.com:443/ab/en/US/adirect/ab?cmd=ABAssayDetailDisplay&assayID=C__27913406_10&Fs=y');) |

# Human genome build 36.3

Abbreviations: SNP, single nucleotide polymorphism; I.D., identification number
