# Supplementary material for: Association of Eleven Common, Low-Penetrance Colorectal Cancer Susceptibility Genetic Variants at Six Risk Loci with Clinical Outcome
Source: PLoS One. 2012 Jul 27;7(7):e41954. doi: 10.1371/journal.pone.0041954 (PMC3407042; doi:10.1371/journal.pone.0041954)
Supplement: Table S2 — Distribution of patients’ clinicopathological characteristics according to genotypes for individual SNP. (DOCX) [file pone.0041954.s002.docx]

**Table S2.** Distribution of patients’ clinicopathological characteristics according to genotypes for individual SNP.#

|  | **rs6983267** | | | | **rs10505477** | | | | **rs7013278** | | | |
| --- | --- | --- | --- | --- | --- | --- | --- | --- | --- | --- | --- | --- |
| **Characteristics** | **All** | **G/G(%)** | **G/T(%)** | **T/T(%)** | **All** | **A/A(%)** | **A/G(%)** | **G/G(%)** | **All** | **C/C(%)** | **C/T(%)** | **T/T(%)** |
| **Age, years** |  |  |  |  |  |  |  |  |  |  |  |  |
| <50 | 73 | 18(24.7) | 40(54.8) | 15(20.6) | 73 | 17(23.3) | 39(53.4) | 17(23.3) | 73 | 31(42.5) | 38(52.1) | 4(5.5) |
| 50-60 | 133 | 59(44.4) | 56(42.1) | 18(13.5) | 133 | 53(39.9) | 60(45.1) | 20(15.0) | 132 | 42(31.8) | 66(50.0) | 24(18.2) |
| >60 | 361 | 138(38.2) | 155(42.9) | 68(18.8) | 358 | 127(35.5) | 160(44.7) | 71(19.8) | 356 | 116(32.6) | 182(51.1) | 58(16.3) |
| *P value* |  |  |  | *0.06* |  |  |  | *0.14* |  |  |  | *0.08* |
| **Gender** |  |  |  |  |  |  |  |  |  |  |  |  |
| Male | 296 | 104(35.1) | 138(46.6) | 54(18.2) | 295 | 95(32.2) | 142(48.1) | 58(19.7) | 292 | 109(37.3) | 142(48.6) | 41(14.0) |
| Female | 271 | 111(41.0) | 113(41.7) | 47(17.3) | 269 | 102(37.9) | 117(43.5) | 50(18.6) | 269 | 80(29.7) | 144(53.5) | 45(16.7) |
| *P value* |  |  |  | *0.35* |  |  |  | *0.36* |  |  |  | *0.16* |
| **Ethnicity** |  |  |  |  |  |  |  |  |  |  |  |  |
| European-American | 462 | 134(29.0) | 229(49.6) | 99(21.4) | 462 | 131(28.4) | 227(49.1) | 104(22.5) | 457 | 166(36.3) | 230(50.3) | 61(13.4) |
| African-American | 105 | 81(77.1) | 22(21.0) | 2(1.9) | 102 | 66(64.7) | 32(31.4) | 4(3.9) | 104 | 23(22.1) | 56(53.9) | 25(24.0) |
| *P value* |  |  |  | *<0.001** |  |  |  | *<0.001** |  |  |  | *0.003** |
| **Stage** |  |  |  |  |  |  |  |  |  |  |  |  |
| 1 | 157 | 58(36.9) | 74(47.1) | 25(15.9) | 156 | 55(35.3) | 74(47.4) | 27(17.3) | 155 | 44(28.4) | 87(56.1) | 24(15.5) |
| 2 | 156 | 59(37.8) | 66(42.3) | 31(19.9) | 155 | 55(35.5) | 66(42.6) | 34(21.9) | 154 | 64(41.6) | 67(43.5) | 23(14.9) |
| 3 | 158 | 61(38.6) | 70(44.3) | 27(17.1) | 158 | 53(33.5) | 77(48.7) | 28(17.7) | 155 | 48(31.0) | 83(53.6) | 24(15.5) |
| 4 | 67 | 23(34.3) | 30(44.8) | 14(20.9) | 67 | 22(32.8) | 30(44.8) | 15(22.4) | 68 | 22(32.4) | 37(54.4) | 9(13.2) |
| *P value* |  |  |  | *0.94* |  |  |  | *0.89* |  |  |  | *0.29* |
| **Tumor site** |  |  |  |  |  |  |  |  |  |  |  |  |
| Colon | 253 | 95(37.6) | 107(42.3) | 51(20.2) | 252 | 88(34.9) | 113(44.8) | 51(20.2) | 247 | 82(33.2) | 126(51.0) | 39(15.8) |
| Rectum | 233 | 87(37.3) | 111(47.6) | 35(15.0) | 232 | 79(34.1) | 113(48.7) | 40(17.4) | 233 | 77(33.1) | 121(51.9) | 35(15.0) |
| *P value* |  |  |  | *0.28* |  |  |  | *0.61* |  |  |  | *0.97* |
| **Treatment** |  |  |  |  |  |  |  |  |  |  |  |  |
| **Surgery** |  |  |  |  |  |  |  |  |  |  |  |  |
| Yes | 557 | 212(38.1) | 245(44.0) | 100(18.0)) | 554 | 194(35.0) | 253(45.7) | 107(19.3) | 551 | 187(33.9) | 279(50.6) | 85(15.4) |
| No | 8 | 1(12.5) | 6(75.0) | 1(12.5) | 8 | 1(12.5) | 6(75.0) | 1(12.5) | 8 | 2(25.0) | 6(75.0) | 0(0.0) |
| *P value* |  |  |  | *0.19* |  |  |  | *0.33* |  |  |  | *0.43* |
| **Chemotherapy** |  |  |  |  |  |  |  |  |  |  |  |  |
| Yes | 291 | 103(35.4) | 132(45.4) | 56(19.2) | 290 | 95(32.8) | 137(47.2) | 58(20.0) | 288 | 100(34.7) | 147(51.0) | 41(14.2) |
| No | 269 | 108(40.2) | 117(43.5) | 44(16.4) | 267 | 99(37.1) | 119(44.6) | 49(18.4) | 266 | 86(32.3) | 136(51.1) | 44(16.5) |
| *P value* |  |  |  | *0.46* |  |  |  | *0.56* |  |  |  | *0.70* |

**Table S2.** Distribution of patients’ clinicopathological characteristics according to genotypes for individual SNP (Continued).#

|  | **rs7014346** | | | | **rs719725** | | | | **rs10795668** | | | |
| --- | --- | --- | --- | --- | --- | --- | --- | --- | --- | --- | --- | --- |
| **Characteristics** | **All** | **G/G(%)** | **A/G(%)** | **A/A(%)** | **All** | **A/A(%)** | **A/C(%)** | **C/C(%)** | **All** | **G/G(%)** | **A/G(%)** | **A/A(%)** |
| **Age, years** |  |  |  |  |  |  |  |  |  |  |  |  |
| <50 | 73 | 33(45.2) | 37(50.7) | 3(4.1) | 72 | 29(40.3) | 34(47.2) | 9(12.5) | 67 | 38(56.7) | 17(25.4) | 12(17.9) |
| 50-60 | 134 | 49(36.6) | 65(48.5) | 20(14.9) | 134 | 63(47.0) | 59(44.0) | 12(9.0) | 131 | 84(64.1) | 37(28.2) | 10(7.6) |
| >60 | 361 | 129(35.7) | 182(50.4) | 50(13.9) | 358 | 146(40.8) | 165(46.1) | 47(13.1) | 348 | 180(51.7) | 141(40.5) | 27(7.8) |
| *P value* |  |  |  | *0.11* |  |  |  | *0.65* |  |  |  | *0.005** |
| **Gender** |  |  |  |  |  |  |  |  |  |  |  |  |
| Male | 297 | 121(40.7) | 139(46.8) | 37(12.5) | 297 | 124(41.8) | 139(46.8) | 34(11.5) | 287 | 156(54.4) | 101(35.2) | 30(10.5) |
| Female | 271 | 90(33.2) | 145(53.5) | 36(13.3) | 267 | 114(42.7) | 119(44.6) | 34(12.7) | 259 | 146(56.4) | 94(36.3) | 19(7.3) |
| *P value* |  |  |  | *0.17* |  |  |  | *0.83* |  |  |  | *0.45* |
| **Ethnicity** |  |  |  |  |  |  |  |  |  |  |  |  |
| European-American | 464 | 171(36.9) | 232(50.0) | 61(13.2) | 460 | 176(38.3) | 224(48.7) | 60(13.0) | 447 | 215(48.1) | 183(40.9) | 49(11.0) |
| African-American | 104 | 40(38.5) | 52(50.0) | 12(11.5) | 104 | 62(59.6) | 34(32.7) | 8(7.7) | 99 | 87(87.9) | 12(12.1) | 0(0.0) |
| *P value* |  |  |  | *0.91* |  |  |  | *<0.001** |  |  |  | *<0.001** |
| **Stage** |  |  |  |  |  |  |  |  |  |  |  |  |
| 1 | 157 | 52(33.1) | 85(54.1) | 20(12.7) | 156 | 53(34.0) | 79(50.6) | 24(15.4) | 155 | 81(52.3) | 62(40.0) | 12(7.7) |
| 2 | 157 | 68(43.3) | 69(44.0) | 20(12.7) | 156 | 67(43.0) | 71(45.5) | 18(11.5) | 148 | 84(56.8) | 49(33.1) | 15(10.1) |
| 3 | 158 | 55(34.8) | 81(51.3) | 22(13.9) | 155 | 76(49.0) | 61(39.4) | 18(11.6) | 149 | 85(57.1) | 49(32.9) | 15(10.1) |
| 4 | 67 | 25(37.3) | 33(49.3) | 9(13.4) | 68 | 28(41.2) | 34(50.0) | 6(8.8) | 66 | 39(59.1) | 23(34.9) | 4(6.1) |
| *P value* |  |  |  | *0.63* |  |  |  | *0.19* |  |  |  | *0.79* |
| **Tumor site** |  |  |  |  |  |  |  |  |  |  |  |  |
| Colon | 253 | 95(37.6) | 123(48.6) | 35(13.8) | 250 | 113(45.2) | 107(42.8) | 30(12.0) | 240 | 124(51.7) | 96(40.0) | 20(8.3) |
| Rectum | 234 | 84(35.9) | 121(51.7) | 29(12.4) | 233 | 88(37.8) | 116(49.8) | 29(12.5) | 227 | 133(58.6) | 71(31.3) | 23(10.1) |
| *P value* |  |  |  | *0.79* |  |  |  | *0.24* |  |  |  | *0.14* |
| **Treatment** |  |  |  |  |  |  |  |  |  |  |  |  |
| **Surgery** |  |  |  |  |  |  |  |  |  |  |  |  |
| Yes | 558 | 208(37.3) | 278(49.8) | 72(12.9) | 554 | 233(42.1) | 255(46.0) | 66(11.9) | 537 | 298(55.5) | 191(35.6) | 48(8.9) |
| No | 8 | 3(37.5) | 5(62.5) | 0(0.0) | 8 | 5(62.5) | 1(12.5) | 2(25.0) | 7 | 3(42.9) | 4(57.1) | 0(0.0) |
| *P value* |  |  |  | *0.79* |  |  |  | *0.08* |  |  |  | *0.60* |
| **Chemotherapy** |  |  |  |  |  |  |  |  |  |  |  |  |
| Yes | 291 | 109(37.5) | 145(49.8) | 37(12.7) | 289 | 127(43.9) | 127(43.9) | 35(12.1) | 277 | 152(54.9) | 97(35.0) | 28(10.1) |
| No | 270 | 99(36.7) | 136(50.4) | 35(13.0) | 268 | 107(39.9) | 129(48.1) | 32(11.9) | 262 | 147(56.1) | 94(35.9) | 21(8.0) |
| *P value* |  |  |  | *0.99* |  |  |  | *0.58* |  |  |  | *0.72* |

**Table S2.** Distribution of patients’ clinicopathological characteristics according to genotypes for individual SNP (Continued).#

|  | **rs3802842** | | | | **rs10318** | | | | **rs4779584** | | | |
| --- | --- | --- | --- | --- | --- | --- | --- | --- | --- | --- | --- | --- |
| **Characteristics** | **All** | **A/A(%)** | **A/C(%)** | **C/C(%)** | **All** | **C/C(%)** | **C/T(%)** | **T/T(%)** | **All** | **C/C(%)** | **C/T(%)** | **T/T(%)** |
| **Age, years** |  |  |  |  |  |  |  |  |  |  |  |  |
| <50 | 73 | 32(43.8) | 28(38.4) | 13(17.8) | 70 | 51(72.9) | 15(21.4) | 4(5.7) | 73 | 38(52.1) | 28(38.4) | 7(9.6) |
| 50-60 | 134 | 59(44.0) | 61(45.5) | 14(10.5) | 132 | 89(67.4) | 40(30.3) | 3(2.3) | 130 | 69(53.1) | 51(39.2) | 10(7.7) |
| >60 | 363 | 170(46.8) | 155(42.7) | 38(10.5) | 354 | 241(68.1) | 102(28.8) | 11(3.1) | 355 | 197(55.5) | 128(36.1) | 30(8.5) |
| *P value* |  |  |  | *0.46* |  |  |  | *0.49* |  |  |  | *0.94* |
| **Gender** |  |  |  |  |  |  |  |  |  |  |  |  |
| Male | 298 | 134(45.0) | 137(46.0) | 27(9.1) | 289 | 200(69.2) | 77(26.6) | 12(4.2) | 294 | 164(55.8) | 109(37.1) | 21(7.1) |
| Female | 272 | 127(46.7) | 107(39.3) | 38(14.0) | 267 | 181(67.8) | 80(30.0) | 6(2.3) | 264 | 140(53.0) | 98(37.1) | 26(9.9) |
| *P value* |  |  |  | *0.10* |  |  |  | *0.35* |  |  |  | *0.50* |
| **Ethnicity** |  |  |  |  |  |  |  |  |  |  |  |  |
| European-American | 465 | 221(47.5) | 196(42.2) | 48(10.3) | 454 | 289(63.7) | 148(32.6) | 17(3.7) | 455 | 281(61.8) | 153(33.6) | 21(4.6) |
| African-American | 105 | 40(38.1) | 48(45.7) | 17(16.2) | 102 | 92(90.2) | 9(8.8) | 1(1.0) | 103 | 23(22.3) | 54(52.4) | 26(25.2) |
| *P value* |  |  |  | *0.10* |  |  |  | *<0.001** |  |  |  | *<0.001** |
| **Stage** |  |  |  |  |  |  |  |  |  |  |  |  |
| 1 | 157 | 83(52.9) | 62(39.5) | 12(7.6) | 154 | 102(66.2) | 43(27.9) | 9(5.8) | 155 | 79(51.0) | 57(36.8) | 19(12.3) |
| 2 | 158 | 72(45.6) | 62(39.2) | 24(15.2) | 153 | 100(65.4) | 50(32.7) | 3(2.0) | 154 | 85(55.2) | 60(39.0) | 9(5.8) |
| 3 | 159 | 71(44.7) | 68(42.8) | 20(12.6) | 154 | 108(70.1) | 43(27.9) | 3(2.0) | 154 | 84(54.6) | 57(37.0) | 13(8.4) |
| 4 | 67 | 22(32.8) | 37(55.2) | 8(11.9) | 66 | 49(74.2) | 14(21.2) | 3(4.6) | 67 | 43(64.2) | 20(29.9) | 4(6.0) |
| *P value* |  |  |  | *0.07* |  |  |  | *0.27* |  |  |  | *0.36* |
| **Tumor site** |  |  |  |  |  |  |  |  |  |  |  |  |
| Colon | 255 | 115(45.1) | 112(43.9) | 28(11.0) | 243 | 164(67.5) | 70(28.8) | 9(3.7) | 249 | 137(55.0) | 87(34.9) | 25(10.0) |
| Rectum | 234 | 109(46.6) | 97(41.5) | 28(12.0) | 232 | 161(69.4) | 63(27.2) | 8(3.5) | 231 | 127(55.0) | 88(38.1) | 16(6.9) |
| *P value* |  |  |  | *0.85* |  |  |  | *0.89* |  |  |  | *0.44* |
| **Treatment** |  |  |  |  |  |  |  |  |  |  |  |  |
| **Surgery** |  |  |  |  |  |  |  |  |  |  |  |  |
| Yes | 560 | 257(45.9) | 238(42.5) | 65(11.6) | 546 | 371(68.0) | 157(28.8) | 18(3.3) | 548 | 294(53.7) | 207(37.8) | 47(8.6) |
| No | 8 | 3(37.5) | 5(62.5) | 0(0.0) | 8 | 8(100.0) | 0(0.0) | 0(0.0) | 8 | 8(100.0) | 0(0.0) | 0(0.0) |
| *P value* |  |  |  | *0.59* |  |  |  | *0.20* |  |  |  | *0.05* |
| **Chemotherapy** |  |  |  |  |  |  |  |  |  |  |  |  |
| Yes | 292 | 126(43.2) | 130(44.5) | 36(12.3) | 288 | 196(68.1) | 82(28.5) | 10(3.5) | 285 | 158(55.4) | 104(36.5) | 23(8.1) |
| No | 271 | 133(49.1) | 111(41.0) | 27(10.0) | 261 | 181(69.4) | 72(27.6) | 8(3.1) | 266 | 143(53.8) | 100(37.6) | 23(8.7) |
| *P value* |  |  |  | *0.34* |  |  |  | *0.93* |  |  |  | *0.92* |

**Table S2.** Distribution of patients’ clinicopathological characteristics according to genotypes for individual SNP (Continued).#

|  | **rs4464148** | | | | **rs4939827** | | | |
| --- | --- | --- | --- | --- | --- | --- | --- | --- |
| **Characteristics** | **All** | **T/T(%)** | **C/T(%)** | **C/C(%)** | **All** | **C/C(%)** | **C/T(%)** | **T/T(%)** |
| **Age, years** |  |  |  |  |  |  |  |  |
| <50 | 72 | 37(51.4) | 27(37.5) | 8(11.1) | 73 | 19(26.0) | 33(45.2) | 21(28.8) |
| 50-60 | 134 | 69(51.5) | 55(41.0) | 10(7.5) | 135 | 38(28.2) | 67(49.6) | 30(22.2) |
| >60 | 355 | 164(46.2) | 159(44.8) | 32(9.0) | 361 | 105(29.1) | 178(49.3) | 78(21.6) |
| *P value* |  |  |  | *0.66* |  |  |  | *0.77* |
| **Gender** |  |  |  |  |  |  |  |  |
| Male | 296 | 150(50.7) | 117(39.5) | 29(9.8) | 298 | 87(29.2) | 145(48.7) | 66(22.2) |
| Female | 265 | 120(45.3) | 124(46.8) | 21(7.9) | 271 | 75(27.7) | 133(49.1) | 63(23.3) |
| *P value* |  |  |  | *0.21* |  |  |  | *0.90* |
| **Ethnicity** |  |  |  |  |  |  |  |  |
| European-American | 456 | 198(43.4) | 214(46.9) | 44(9.7) | 463 | 113(24.4) | 235(50.8) | 115(24.8) |
| African-American | 105 | 72(68.6) | 27(25.7) | 6(5.7) | 106 | 49(46.2) | 43(40.6) | 14(13.2) |
| *P value* |  |  |  | *<0.001** |  |  |  | *<0.001** |
| **Stage** |  |  |  |  |  |  |  |  |
| 1 | 154 | 65(42.2) | 74(48.1) | 15(9.7) | 156 | 45(28.9) | 76(48.7) | 35(22.4) |
| 2 | 155 | 66(42.6) | 69(44.5) | 20(12.9) | 157 | 36(22.9) | 67(42.7) | 54(34.4) |
| 3 | 156 | 90(57.7) | 58(37.2) | 8(5.1) | 161 | 54(33.5) | 86(53.4) | 21(13.0) |
| 4 | 67 | 34(50.8) | 29(43.3) | 4(6.0) | 67 | 18(26.9) | 36(53.7) | 13(19.4) |
| *P value* |  |  |  | *0.04** |  |  |  | *0.001** |
| **Tumor site** |  |  |  |  |  |  |  |  |
| Colon | 249 | 121(48.6) | 108(43.4) | 20(8.0) | 254 | 70(27.6) | 128(50.4) | 56(22.1) |
| Rectum | 231 | 110(47.6) | 99(42.9) | 22(9.5) | 236 | 73(30.9) | 111(47.0) | 52(22.0) |
| *P value* |  |  |  | *0.86* |  |  |  | *0.70* |
| **Treatment** |  |  |  |  |  |  |  |  |
| **Surgery** |  |  |  |  |  |  |  |  |
| Yes | 551 | 263(47.7) | 238(43.2) | 50(9.1) | 559 | 159(28.4) | 272(48.7) | 128(22.9) |
| No | 8 | 6(75.0) | 2(25.0) | 0(0.0) | 8 | 2(25.0) | 6(75.0) | 0(0.0) |
| *P value* |  |  |  | *0.39* |  |  |  | *0.26* |
| **Chemotherapy** |  |  |  |  |  |  |  |  |
| Yes | 288 | 149(51.7) | 119(41.3) | 20(6.9) | 292 | 82(28.1) | 141(48.3) | 69(23.6) |
| No | 266 | 118(44.4) | 120(45.1) | 28(10.5) | 270 | 78(28.9) | 136(50.4) | 56(20.7) |
| *P value* |  |  |  | *0.13* |  |  |  | *0.72* |

**Footnotes for Table S2:** # Because of missing values and genotyping failure, total n for each SNP does not always tally to 589; Percentages may not always add up to 100% because numbers were rounded; * Statistically significant p value based on Fisher’s exact test at *P*<0.05
